# Photolithography‐Compatible Templated Patterning of Functional Organic Materials in Emulsion

**DOI:** 10.1002/advs.201500304

**Published:** 2015-12-11

**Authors:** Kai Zhang, Xiaoyong Deng, Qun Fu, Yun Meng, Huaping Zhao, Wenchong Wang, Minghong Wu, Yong Lei

**Affiliations:** ^1^ Institute of Nanochemistry and NanobiologySchool of Environmental and Chemical EngineeringShanghai University99 Shangda RoadShanghai200444P.R. China; ^2^ Institut für Physik & IMN MacroNano (ZIK)Technische Universität IlmenauIlmenau98693Germany; ^3^ Shanghai Applied Radiation InstituteShanghai UniversityShanghai200444P.R. China

**Keywords:** emulsion, patterning, organic semiconductors

## Abstract

**A solution‐processed patterning of functional organic materials in emulsion** is reported. The concept is to absorb microdrops onto predefined locations by hydrophilicity difference. Owing to a universal solvent used, the method can be applied to pattern variety materials on substrates of interest over large size. The figure shows Rhodamine 6G patterned film.

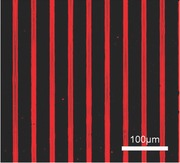

Functional organic materials are the carbon‐based molecules with advanced optic and/or electronic properties.^1^, ^2^ Owing to the extensive applications such as organic electronics and dye labeling, the materials have attracted much attention in last three decades.^3^, ^4^ In many cases, particularly in organic electronics, patterning of the organic materials is a crucial step for improving device performances and integration level.^5^ Ideally, the patterning technique requires high resolution and high yield over large area. Unfortunately, direct applying of photolithography leads to both damage of functionalities of the materials and degradation of device performances. Alternatively, shadow mask, printing, ink‐jet writing, and capsulation assisted photolithography are proposed and demonstrated.^6^, ^7^, ^8^, ^9^, ^10^, ^11^, ^12^, ^13^, ^14^, ^15^, ^16^ However, these approaches suffer variously from insufficient resolution, low yield, and poor scalability. Recently, a top‐down assisted bottom‐up strategy, template directed patterning, was proposed. The strategy utilizes prepatterns as nucleation locations for diffusion molecules in vacuum and flow channels for molten molecules in air.^17^, ^18^, ^19^, ^20^ However, the former approach involves complicated growth dynamics in vacuum; the later one is limited to continuous patterns. Moreover, the scalability to large area is still a big challenge for the both template approaches owing to sample holder size limitation in vacuum chamber and saturation of flow velocity of molten molecule liquid in air. In this communication, we demonstrate a facile way to selective absorption microdroplets onto predefined locations in emulsion, resulting in micropatterning of functional organic materials on surfaces of interest.

Processing organic materials in solution is desirable to fully explore low‐cost large‐area organic electronics. Efforts for fabricating organic field effect transistors in solution have been reported by dewetting and controlled crystallization.^21^ However, the device dimensions must be over hundreds of micrometers to isolate each single device. The challenge lies on difficulty to separate materials to be patterned into micrometer scale. It is well known that emulsion can be formed when agitating mixture of two immiscible liquids. The emulsion contains micro/nanodroplets with size ranging from nanometers to tens of micrometers.^22^ The concept of the study is to absorb the functional material containing micro/nanodroplets selectively onto predefined locations, resulting in patterned films after evaporation of liquid droplet (schematically shown in **Figure** **1**). In the study, we first added dyeddimethyl sulfoxide (DMSO) solution to hexane (Figure 1a), and then agitated the mixture by ultrasonic to create emulsion (Figure 1b). Then Au patterned Octadecyltrichlorosilane (OTS) modified SiO_2_ templates (Figure 1c), which were prepared by standard photolithography as shown in Figure 1d, were immersed into the DMSO/hexane emulsion for several minutes to absorb the dyed DMSO droplets onto Au (Figure 1e). Finally, the samples were taken out the emulsion and dried to form patterned dye films on the Au, as shown in Figure 1f.

**Figure 1:**
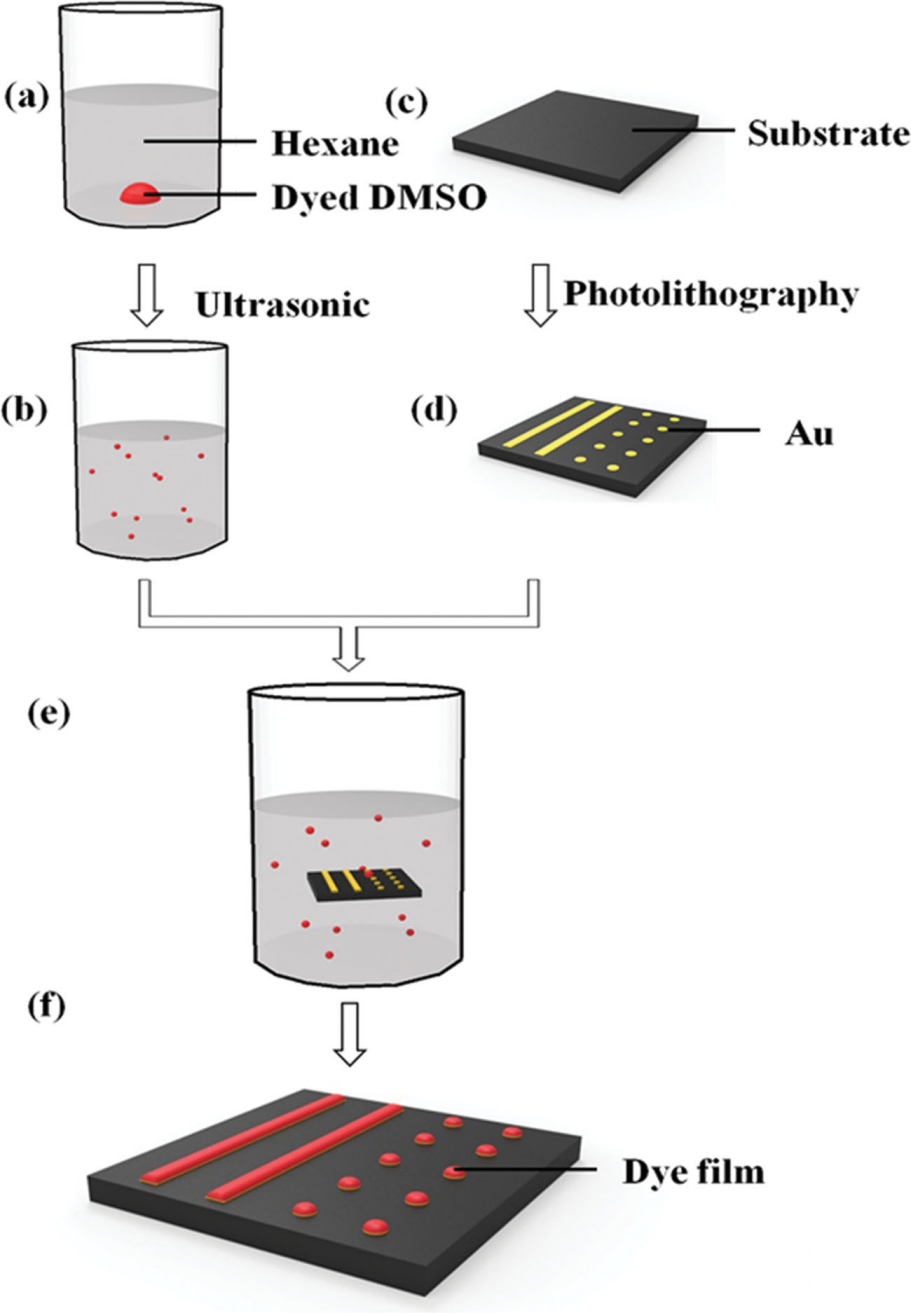
Schematic representation of template patterning of functional organic materials in emulsion. a) Mixture of two immiscible liquids, b) ultrasonic treatment of the mixture to form emulsion, c) OTS modification of SiO_2_, d) patterning of substrate by standard photolithography, e) immersion of the templates into the emulsions, and f) formation of patterned dye films after evaporation of solvent.

**Figure** **2**a,b shows photographs of Rhodamine 6G dyed DMSO/hexane mixture before and after ultrasonication. When mixed together, the dyed DMSO goes to the bottom of hexane owing to the high density, shown in Figure 2a in red color. After ultrasonication, the mixture turned to red, indicating dispersion of DMSO in hexane and formation of emulsion shown in Figure 2b. Typically, the size of the DMSO droplets in emulsion is in micrometers.^23^ Dynamics studies show that the emulsion exhibits a certain stability in hours which can be further improved by the presence of surfactant at interface.^24^ In the experiments, we directly apply DMSO/hexane without surfactant because the stability duration is long enough to absorb droplets onto template. We choose DMSO because it is hydrophilic to Au with a contact angle *θ* of 43°, and the contact angle increases to 67° on OTS/Si, shown in Figure 2c,d. In addition, DMSO is a “universal” solvent that can solubilize a wide variety of otherwise poorly soluble organic materials.^25^

**Figure 2:**
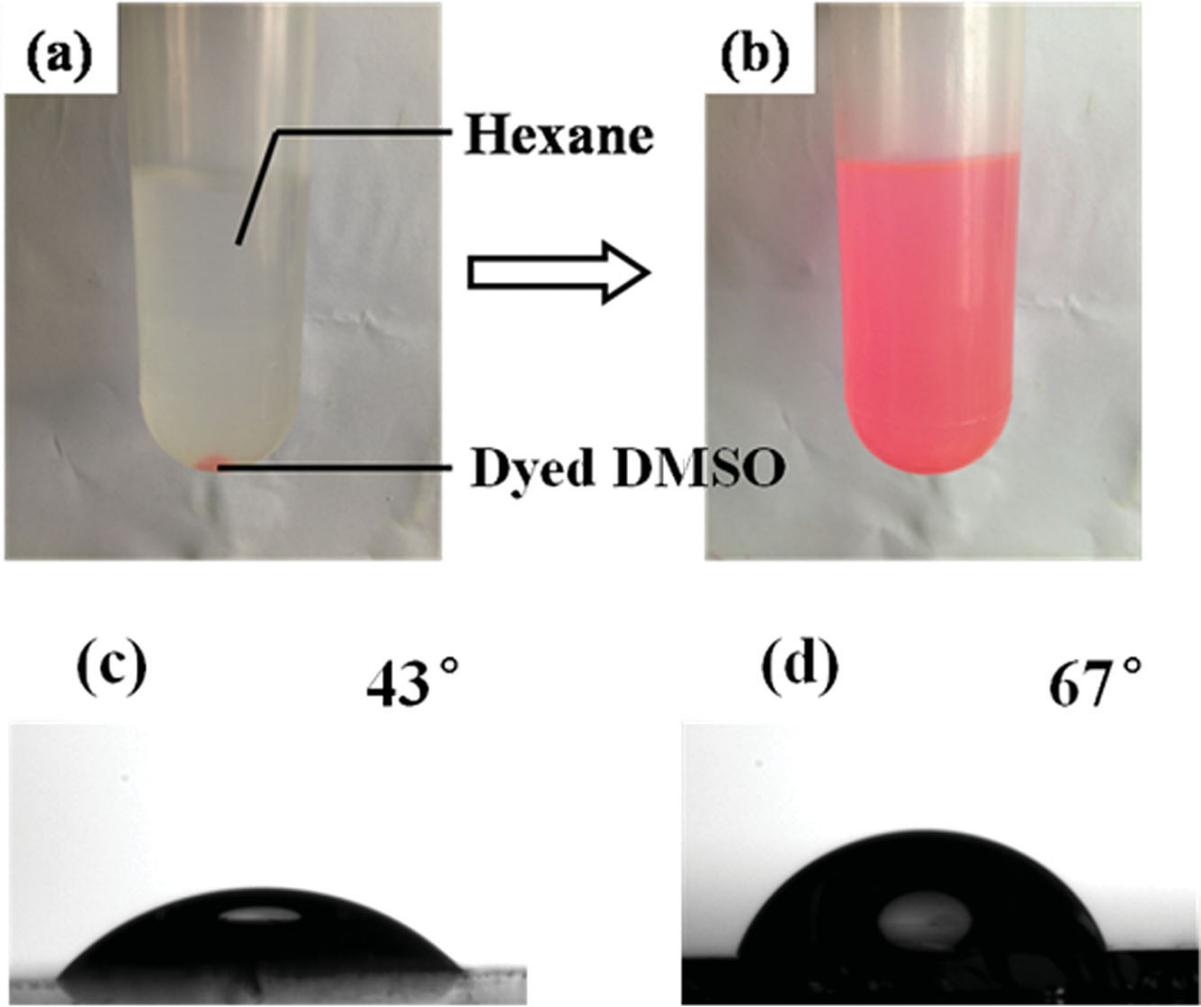
Photographs of Rhodamine 6G dyed DMSO/hexane mixture a) before and b) after ultrasonication, and contact angle of DMSO on c) Au and d) OTS modified Si.

**Figure** **3** shows video snaps of the driving of Rodamine 6G dyed DMSO liquid drop from OTS/Si to Au by the difference in hydrophilicity. The experiment was performed on an OTS/Si surface which is half covered by Au. When a DMSO drop was dipped onto the OTS/Si surface, it stabilized on the surface with the contact angle of 68° (Figure 3a). We pulled the drop toward Au by a tip. When contacting with the Au edge, the DMSO drop moved to the Au in 1 s (Figure 3b). Finally, the drop completely resides on Au with the contact angle of 43° (Figure 3c).

**Figure 3:**
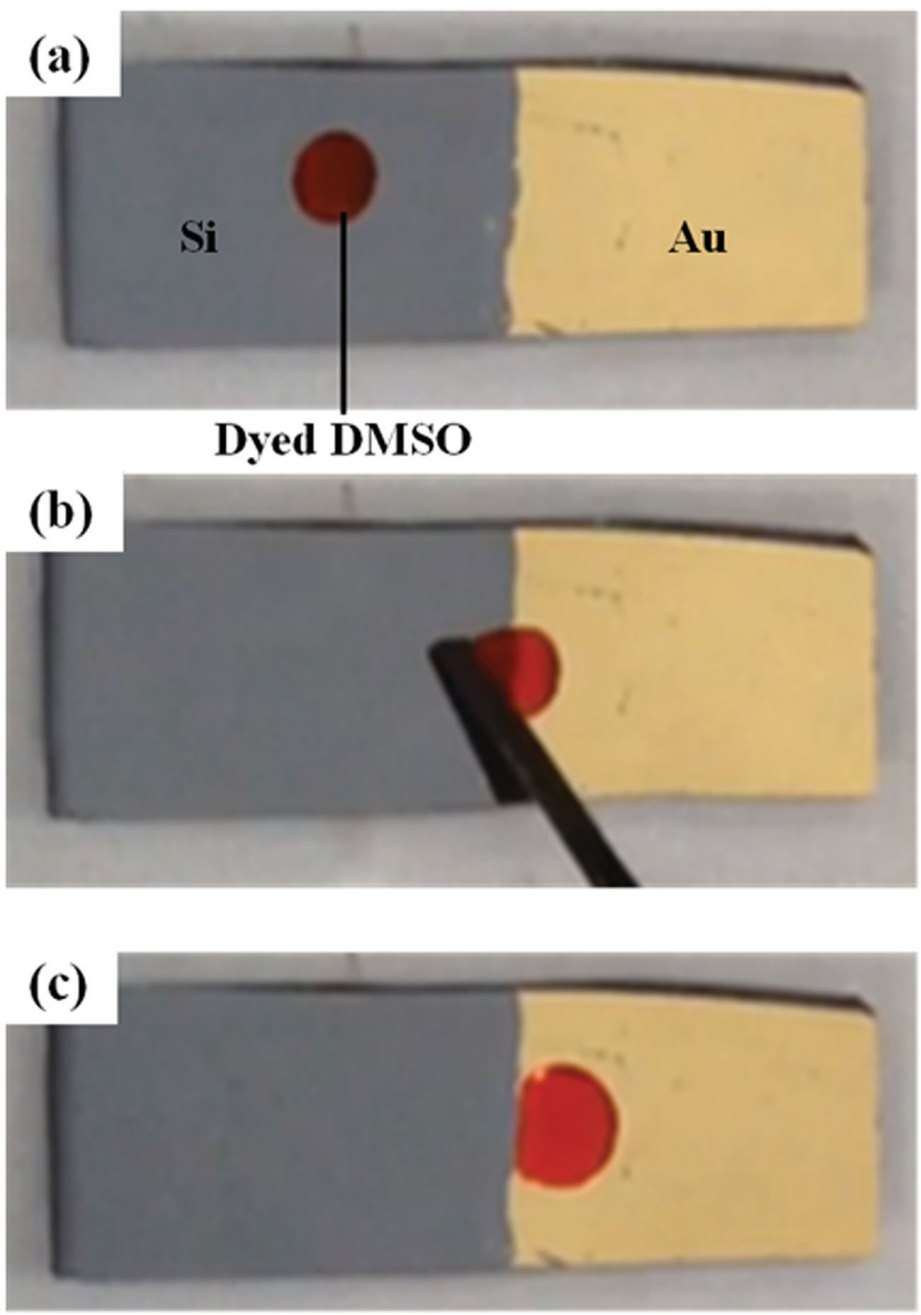
Video snaps of Rhodamine 6G dyed DMSO driving from OTS/Si to Au, with the drop a) on OTS/Si, b) contacting Au edge, and c) on Au.

Similar to selective absorption of DMSO drop on Au as demonstrated in Figure 3, the DMSO droplets in the emulsion can also be attached to micropatterns. **Figure** **4**a shows fluorescence microscope image of the line patterned Rhodamine 6G films fabricated by the procedure shown in Figure 1. The concentration of the Rhodamine 6G in DMSO is 1.0 wt%, the DMSO was mixed into hexane with a volume ratio of 4:1000, and the immersion time is 10 min, respectively. The image shows strong red color on the Au stripes, indicating the presence of Rhodamine 6G. We further performed photoluminescence (PL) spectra on both Rhodamine 6G dyed DMSO (Figure 2b) and patterned Rohodamine film (Figure 4a), as shown in Figure 4b. The PL spectrum of Rhodamine 6G in DMSO shows a typical red emission with a peak at 622 nm.^26^ The emission is redshifted to 639 nm owing to molecular interaction by aggregation, confirming successful confinement of Rhodamine 6G on predefined areas.^19^ The absorption of DMSO microdroplets onto Au further enables us to tune the PL intensity of Rhodamine 6G by simply varying the concentration. Figure 4c shows the PL intensity with Rhodamine 6G concentration from 0.75 to 1.75 wt%. The immersion time was kept by 10 min for all the samples, and the intensity was calculated by averaging 50 lines in one image filed. A linear increase of PL intensity with Rhodamine 6G concentration in DMSO is achieved, indicating that the amount of dye molecules can be controlled by concentration. We note that the PL intensity can also be tuned by changing the immersion time of samples in the emulsion, providing another way for the amount control.

**Figure 4:**
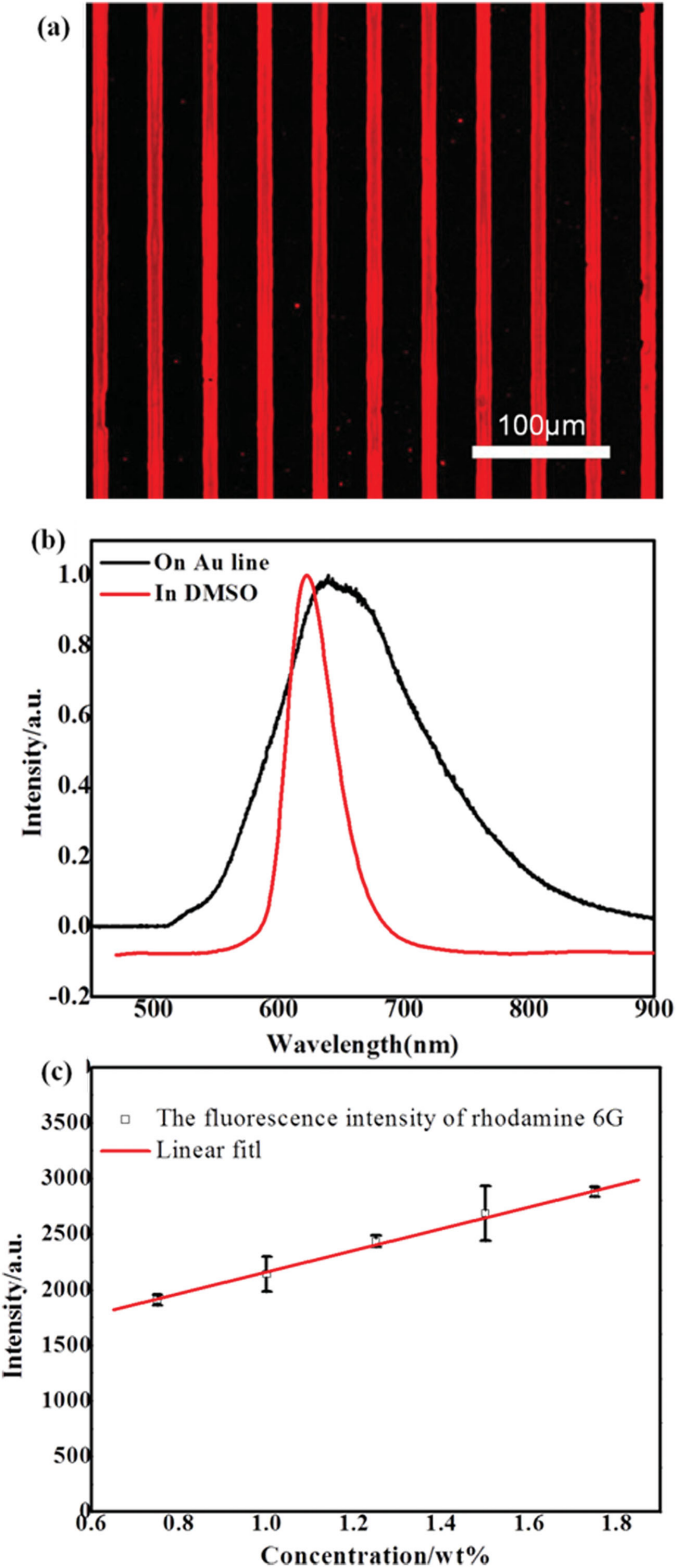
a) Fluorescence microscope image of Rhodamine 6G on 10 μm Au line patterned Si. b) normalized PL spectra of 1.0 wt% Rhodamine 6G in DMSO and on Au stripes, and c) PL intensity of Rhodamine 6G with different concentration in DMSO.

Owing to the confinement of the droplets onto the pre‐defined locations, the strategy enables us to fabricate various functional organic materials patterns with different shapes on substrates of interests, as shown in **Figure** **5**. For a more broad interest, we use Aluminium‐tris(8‐hydroxychinolin), Alq3 which is a light emissive molecule that is widely used in OLEDs.^27^ Figure 5a shows fluorescence microscope image of Alq3 on 4 μm Au dots with the procedure. The solution process can be applied to pattern functional organic material (Rhodamine 6G here in Figure 5b,c) on flexible polyethylene terephthalate with 20 μm Au square patterned by shadow mask. The technique can easily be extended to substrates of interest over larger size, with the resolution down to micrometers which is comparable to inkjet printing.^28^ In addition, the confinement of the drops in micrometers can suppress the “coffee ring” effect during the solvent evaporation, leading to uniform films on pre‐defined areas as shown in Figure 5a,c.^29^

**Figure 5:**
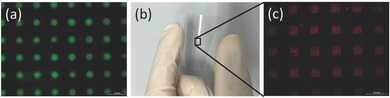
a) Fluorescence microscope images of Alq3 on 4 μm Au dots patterned OTS/Si, b) photography, and c) fluorescence microscope images of Rhodamine 6G on 20 μm Au square patterned PET substrate.

In conclusion, we demonstrated a facile method to pattern functional organic materials in emulsion, overcoming the size limitation of template directed strategy either by vacuum deposition or surface microfluidics. The method is completely compatible with photolithography on substrates of interest, with the resolution down to micrometers for the application like organic microdisplays. The concept, which is based on wettability control of templates and substrates, can be extended to organic monolayers for hydrophilic/hydrophobic modifications.^30^ We believe the resolution can be further improved when using template in nanometers and nanoemulsions.

## Experimental Section

*Substrate Preparation*: After sequential ultrasonic rinsed in chloroform, acetone, ethanol, and deionized water each for 10 min, the silicon substrates were dried by N_2_ flow for OTS modification. The modification was done by immersing the Si substrates into an OTS:Chloroform:Hexane (1:1200:2800 in volume) mixture for 5 h.

*Template Fabrication*: The OTS modified Si substrates were processed by standard photoresist spin coating, baking, UV exposure through a shadow mask, and developing. After the pattern formation on photoresist, the samples were coated with a 3 nm Ti adhesion layer and 20 nm Au layer by thermal evaporation, followed by lift off and transferring the patterns onto OTS/Si surfaces.

*Pattern Formation and Characterization*: The DMSO (40 μL) solution was added to hexane (10 mL), and then the phase‐separated DMSO/hexane mixture was ultrasonicated (40 kHz for 60 s to form DMSO/hexane emulsion. Then the Au patterned OTS/Si substrates were immersed in the emulsion for 5 min, then taken out and heated for 5 min at 60 °C, to evaporate solvent. Finally, the samples were cooled to room temperature for Fluorescence microscope and PL measurements.
